# Up‐regulation of FoxO1 contributes to adverse vascular remodelling in type 1 diabetic rats

**DOI:** 10.1111/jcmm.15935

**Published:** 2020-10-27

**Authors:** Jingjin Liu, Xiang Xie, Dan Yan, Yongshun Wang, Hongbin Yuan, Yin Cai, Jierong Luo, Aimin Xu, Yu Huang, Chi Wai Cheung, Michael G. Irwin, Zhengyuan Xia

**Affiliations:** ^1^ Department of Anesthesiology University of Hong Kong Hong Kong China; ^2^ Department of Anesthesiology The Second Affiliated Hospital and Yuying Children's Hospital Wenzhou Medical University Wenzhou China; ^3^ Department of Biomedical Science University of Hong Kong Hong Kong China; ^4^ Department of Anesthesiology Changzheng Hospital Second Military Medical University Shanghai China; ^5^ Department of Anesthesiology Guangzhou First People's Hospital Guangzhou Medical University Guangzhou China; ^6^ State Key Laboratory of Pharmaceutical Biotechnology the University of Hong Kong Hong Kong China; ^7^ Heart and Vascular Institute and School of Biomedical Sciences Chinese University of Hong Kong Hong Kong SAR China

**Keywords:** cardiovascular diseases, diabetes, Forkhead box protein O1, vascular remodeling

## Abstract

Vascular complications from diabetes often result in poor outcomes for patients, even after optimized interventions. Forkhead box protein O1 (FoxO1) is a key regulator of cellular metabolism and plays an important role in vessel formation and maturation. Alterations of FoxO1 occur in the cardiovascular system in diabetes, yet the role of FoxO1 in diabetic vascular complications is poorly understood. In Streptozotocin (STZ)‐induced type 1 diabetic rats, FoxO1 expression was up‐regulated in carotid arteries at 8 weeks of diabetes that was accompanied with adverse vascular remodelling characterized as increased wall thickness, carotid medial cross‐sectional area, media‐to‐lumen ratio and decreased carotid artery lumen area. This adverse vascular remodelling induced by hyperglycaemia in diabetic rats required FoxO1 activation as pharmacological inhibition of FoxO1 with 50mg/kg AS1842856 (AS) reversed vascular remodelling in type 1 diabetic rats. The adverse vascular remodelling in type 1 diabetes mellitus (T1DM) occurred concomitantly with increases in pro‐inflammatory factors, adhesion factors, apoptosis, NOD‐like receptor family protein‐3 inflammasome activation and the phenotypic switch of arterial smooth muscle cells, which were all reversed by AS. In addition, FoxO1 inhibition counteracted the down‐regulation of its upstream mediator PDK1 in T1DM. PDK1 activator reduced FoxO1 nuclear translocation, which serves as the basis for subsequent transcriptional regulation during hyperglycaemia. Taken together, our data suggest that FoxO1 is a critical trigger for type 1 diabetes‐induced vascular remodelling in rats, and inhibition of FoxO1 thus offers a potential therapeutic option for diabetes‐associated cardiovascular diseases.

**Abbreviations**
ASC: apoptosis‐associated speck‐like protein with a caspase‐recruitment domain
CVD: cardiovascular disease
FBS: foetal bovine serum
FoxO1: forkhead transcription factor 1
HASMCs: human aorta smooth muscle cells
HG: high glucose
MMP2: matrix metalloproteinases 2
MMP9: matrix metalloproteinases 9
NLRP3: NOD‐like receptor family, pyrin domain containing 3
PDK1: phosphoinositide‐dependent kinase
ROS: reactive oxidative species
TNF‐α: tumour necrosis factor α
VSMCs: vascular smooth muscle cells

## 1 INTRODUCTION

Diabetic vascular complications result in poor outcomes in diabetic patients, even after interventions from all medical disciplines, thereby posing a major challenge for today's healthcare practitioners. However, the precise molecular and cellular mechanisms underlying the pathogenesis of diabetic vascular complications are still not fully understood.

Hyperactivation of forkhead box‐containing protein O subfamily (FoxOs) has been reported to be associated with overt diabetes hallmarks, such as hyperglycaemia, hypertriglyceridaemia and insulin resistance, as well as diabetic complications.^1^, ^2^, ^3^, ^4^, ^5^, ^6^ Forkhead box protein O1 (FoxO1), a member of forkhead box‐containing protein O subfamily, is a transcription factor and acts in a cell‐specific manner to modulate the genes that control gluconeogenesis,^7^, ^8^ blood vessel assembly,^9^ muscle wasting^10^ and adipocyte differentiation.^11^ Its transcriptional activity is dependent on its phosphorylation state.^12^ FoxO1 overexpression in liver and pancreatic β‐cells is sufficient to induce diabetes in mice.^1^ Conversely, FoxO1 gene haploinsufficiency rescues the diabetic phenotype of insulin‐resistant mice through lowering hepatic expression of gluconeogenic enzymes and increasing adipocyte expression of insulin‐sensitizing genes.^1^ Diabetic complications, such as retinopathy and impaired fracture healing, have been linked to elevated FoxO1 transcriptional activity under hyperglycaemic conditions.^4^, ^13^, ^14^, ^15^, ^16^ Based on these observations, we postulated that FoxO1 may be involved in diabetic vascular remodelling pathogenesis.

Vascular remodelling is an active process of structural alteration, depending on a dynamic interaction among locally generated growth factors, vasoactive substances and hemodynamic stimuli.^17^, ^18^ High‐glucose condition directly impairs vascular smooth muscle cells (VSMCs) biology via promoting neo‐intimal hyperplasia, yielding arterial narrowing or occlusion. This pathological process involves matrix production, inflammatory cell infiltration and smooth muscle cells (SMCs) phenotypic switching.^19^ As extracellular matrix scaffold degradation enables tissue reshaping, the participation of specialized enzymes, called matrix metalloproteinases (MMPs), has become the object of intense interest in relation to vascular remodelling.^20^ As demonstrated in early post‐mortem studies, inflammation and apoptotic SMCs play a great role in the progression of artery complications in diabetic patients of sudden coronary death.^21^ Increased apoptotic SMCs in atherosclerotic lesions, in turn, increases the propensity of plaque rupture.^22^ Therefore, the progress of adverse diabetic vascular remodelling involves SMCs apoptosis, MMP expression as well as inflammatory cell infiltration and SMCs phenotypic switching.

3‐phosphoinositide‐dependent protein kinase 1 (PDK1) activates a group of protein kinases belonging to the protein kinase A (PKA)/PKG/PKC kinase family that play important roles in mediating diverse biological processes including vascular function.^23^, ^24^, ^25^, ^26^ Indeed, it has been found that endothelial cells‐specific deletion of PDK1 enhances insulin sensitivity via reducing visceral fat and suppressing angiogenesis.^27^ In addition, PDK1/FoxO1 pathway is important in regulating glucose and energy homeostasis, but little is known about its role in diabetic vascular remodelling.

This study was designed to shed light on the role of FoxO1 in diabetic vasculature alteration, as well as the underlying mechanisms. We showed that elevation of FoxO1 was a crucial factor associated with the increase of pro‐inflammatory factors, MMP expression and SMCs phenotype switching, resulting in the development of pathological vascular remodelling in type 1 diabetes mellitus (T1DM). Subsequently, we demonstrated that FoxO1 inhibition reversed the phenotypic switch of SMCs and lowered the chronic inflammatory states, thereby preventing diabetes‐driven tunica media dysfunction. Inhibition of FoxO1 may provide a valuable tool to alleviate the adverse vascular remodelling in T1DM progression.

## 2 MATERIAL AND METHODS

### 2.1 Induction of diabetes

All rats (male Sprague‐Dawley rats, 250 ± 10g, 6‐8 weeks) were obtained from the Laboratory Animal Service Center (University of Hong Kong). The rats were housed in an animal unit and had free access to standard water and rat chow. The investigation operated in accordance with the procedures described in the Use of Laboratory Animals and Guide for the Care published by the United States National Institutes of Health (NIH Publication No. 85‐23, revised 1996). The experimental protocol applied in this study was supported by the Committee for Use of Live Animals in Teaching and Research (CULATR 4554‐17) of the University of Hong Kong. Diabetes was induced by Streptozotocin (STZ) (Sigma‐Aldrich, St. Louis, MO) tail vein injection at 65 mg/kg in 0.1 M citrate buffer (pH 4.5), or citrate buffer alone as control, under anaesthesia comprised of a combination of ketamine at 67.7 mg/kg bodyweight and xylazine at 6.77 mg/kg. After 72 hours from injection, blood glucose was measured using a One Touch Ultra Glucose meter (Life Scan Inc USA); rats with blood glucose levels >15 mM were recognized as diabetic.

### 2.2 Experimental procedure

Diabetic rats (n = 6) were treated with a selective FoxO1 inhibitor AS1842856 (AS), which has an IC50 of 0.033 mM to inhibit FoxO1, and can block FoxO1 expression at a final concentration of 0.05‐1 mM without showing cytotoxicity.^28^, ^29^ At fourth week of diabetes induction, diabetic rats were administrated intragastrically with AS AS1842856 (50 mg/kg) or the same dose of β‐cyclodextrin as control, twice daily with 12‐hour intervals between each administration, for 8 days before termination. Before administered to animals, the drug AS1842856 was dissolved in 10% w/v β‐cyclodextrin and underwent ultrasound homogenization until obtaining homogenous suspension liquid.^16^ The rats were sacrificed at 8 weeks after diabetes induction. Carotid arteries were then sampled. β‐cyclodextrin has been widely used as solvent control of many pharmaceutical molecules as it increased the biocompatibility of the effect and also help release in a slow way. Our preliminary study showed that β‐cyclodextrin did not affect target protein levels in liver/plasma.^30^

### 2.3 Frozen section preparation

Fresh vascular tissues were embedded in OCT compound and subsequently stored at −80°C. Sections at 8 µm thick were then cut and mounted on gelatin‐coated slides. Before staining, slides were warmed at room temperature for 30‐60 minutes, fixed in ice cold acetone as fixative for 10‐15 minutes and then dried in air at room temperature for 30‐60 minutes. After drying, slides were washed in PBS and subjected to standard staining procedure for immunofluorescence, as well as haematoxylin and eosin (HE) staining.

### 2.4 H&E staining

Sample slides were stained with haematoxylin (H3136, Sigma) for 5 minutes; colour was checked under the microscope. Slides were then washed with tap water 3 times, followed by bluing in Scott's tap water (S5134, Sigma) for 30 seconds. After another 3 times of washing with tap water, slides were counterstained with Eosin Y (230 251, Sigma) for 40 seconds, followed by washing with tap water for another 3 times. After the final wash, slides were mounted with mountant (06 522, Sigma), covered with coverslip, dried overnight and checked with a microscope connected with computer using Olympus cellSens software (Version 2.1).

### 2.5 Immunofluorescence staining

Vascular tissue slides were perforated by 0.1% triton (X100, Sigma) for 15 minutes and then incubated with blocking solution (10% normal goat serum in PBS diluent) for 1 hour. The slides were then washed with PBS for 5 minutes, and slices were incubated with mouse anti‐α‐SMA antibody (1:500; Abcam), ICAM‐1 (1:500; Abcam) or VCAM‐1(1:500; Abcam) mixed with rabbit anti‐FoxO1 antibody (1:100; Cell Signaling Technology) at 4 °C overnight. Primary antibodies were then poured away and washed with PBS 3 times, 10 minutes for each wash. Secondary antibodies (1:1000, Goat anti‐mouse Alexa‐568 and Goat anti‐rabbit EGFP‐488; Cell Signaling Technology). were added and incubated for 1 hour followed by washing with PBS again 3 times, for 10 minutes each time. DAPI (D9542, Sigma) was added for 20 minutes, followed by another 3 times of PBS washing, each time for 5 minutes. Slides were then mounted with mountant (06 522, Sigma), covered with coverslip, dried overnight and checked with a microscope connected with computer using ZEN 2012 (Version 2.3).

### 2.6 Index of vascular structure

The artery wall was observed using a microscope (Leica, Wetzlar, Germany). Mean wall thickness, media‐to‐lumen ratio (M/L ratio), cross‐sectional area (CSA) and lumen area were calculated using Image J.

### 2.7 Cell culture and treatment

The human aortic smooth muscle cells (HASMCs) were obtained from American Type Culture Collection (ATCC). Cells (passage number 10 or less) were cultured in culture medium (Ham's F‐12K, Kaighn's, 21 127 022, Thermo Fisher) with 10% Foetal bovine serum (10099‐141, Thermo Fisher), 1% penicillin/streptomycin (100 U/mL, 15 140 122, Thermo Fisher) and 1% Insulin‐Transferrin‐Selenium (41400‐045, Invitrogen). All cells were incubated at 37°C with air atmosphere containing 5% CO_2_‐95% O_2_.

To determine the specific role of PDK1 in cytoplasmic and nuclear FoxO1 regulation, 5 μM PS48 (PDK1 Activator, Sigma‐Aldrich) as previous report^31^ was applied following high‐glucose (HG, 25 mmol/L) treatment.

### 2.8 Nuclear and cytoplasmic protein separation

Frozen tissues were suspended in a buffer containing 10 mM Tris, pH 7.5, 1.5 mM MgCl_2_, 10 mM KCl and 0.1% Triton X‐100, and lysed by homogenization. Nuclei were recovered by micro‐centrifugation at 17 000 *g* for 5 min. The supernatant, containing cytoplasmic and membrane protein, was collected and stored at −80°C for further Western blot analysis. Nuclear proteins were extracted at 4°C by gently re‐suspending the nuclei pellet in buffer containing 20 mM Tris, pH 7.5, 20% glycerol, 1.5 mM MgCl_2_, 420 mM NaCl, 0.2 mM EDTA and 0.1% Triton X‐100, followed by 1 hour incubation at 4^o^C with occasional vortexing. After micro‐centrifugation at 13,000 x g for 15 minutes at 4°C, the supernatant containing the nuclear protein was collected.

### 2.9 Real‐time polymerase chain reaction

Total RNA was isolated using TRIzol. Equal amounts of RNA were reverse‐transcribed and processed using the PrimeScript RT Master Mix Kit (Cat. number RR036Q, Takara, Shuzou, Japan), according to the manufacturer's instructions. Quantitative real‐time PCR was performed as described^32^ using SYBR Green PCT master mix (RR820A, Takara) on an Applied Biosystems Prism 7000 sequence detection system (Applied Biosystems, Foster City, CA, USA). Amplification conditions were 30 seconds at 95°C for denaturation, 40 cycles of 5 seconds at 95°C and 30 seconds at 60°C. Gene‐specific primers sequences used were listed in Table S1. mRNA levels of the different genes tested were normalized to those of β‐actin.

### 2.10 Western blotting

Protein concentration of lysates was measured with absorption of Coomassie Brilliant Blue in the spectrophotometer. Specific protein levels were assessed by Western Blot as described.^33^ Briefly, equal quantities of protein were separated by SDS‐PAGE and transferred to polyvinylidene difluoride membranes (PVDF, Millipore, Bedford, MA, USA). The membranes were blocked in 5% non‐fat dry milk diluted with Tris Buffered Saline with Tween‐20 (TBST) (in mM: Tris‐HCl 20, NaCl 150, pH 7.5, 0.1% Tween 20) at room temperature for 1 hour, and then probed with antibodies against FoxO1 (1:1000, Cat.2880, Cell Signaling Technology); phosphorylated (p)‐FoxO1 (1:1,000; Cat.9461, Cell Signaling Technology); ICAM‐1 (1:1000; Cat.67836, Cell Signaling Technology); VCAM‐1 (1:1000; Cat.39036, Cell Signaling Technology); NFκB‐p50 (1:1000; Cat.13586); α‐smooth muscle actin (α‐SMA, 1:1000; Cat.19245, Cell Signaling Technology); smooth muscle myosin heavy chain (SM‐MHC, 1:1000, Cat.8505, Cell Signaling Technology); calponin‐1 (1:1000; Cat.17819, Cell Signaling Technology); NLRP3 (1:1000; Cat.13158, Cell Signaling Technology); ASC (1:1000, Cat.13833, Cell Signaling Technology); Bcl‐2 (1:1000; Cat.15071, Cell Signaling Technology); BaX (1:1000, Cat.5023, Cell Signaling Technology); PDK1 (1:1000; Cat.5662, Cell Signaling Technology); caspase‐3 (1:1000; Cat.9662, Cell Signaling Technology); MMP‐2 (1:1000; Cat.92536, Abcam); MMP‐9 (1:1000; Cat76003, Cell Signaling Technology); and GAPDH (1:1000; Cat. 9485, Abcam, and histone (1:1000; Cat.1791, Abcam) at 4°C overnight. After extensive washing, the membranes were incubated with secondary horseradish peroxidase‐conjugated anti‐mouse or anti‐rabbit antibodies (diluted in 1:2000 5% Bovine Serum Albumin solution; Amersham Biosciences, UK). Immunoblots were visualized using an enhanced chemiluminescence detection system (Amersham Pharmacia Biotech, Uppsala, Sweden).

### 2.11 Statistics

For statistical analysis, SPSS version 25.0 software was used. Results were presented as mean ± standard deviation (SD). Comparison among groups was performed using one‐way analysis of variance (ANOVA) followed by Duncan's multiple comparison *post hoc* tests. Differences were considered statistically significant at *P* < .05.

## 3 RESULTS

### 3.1 FoxO1 was elevated in the carotid arteries of diabetic rats that was attributable to adverse vascular remodelling in diabetes

**FIGURE 1:**
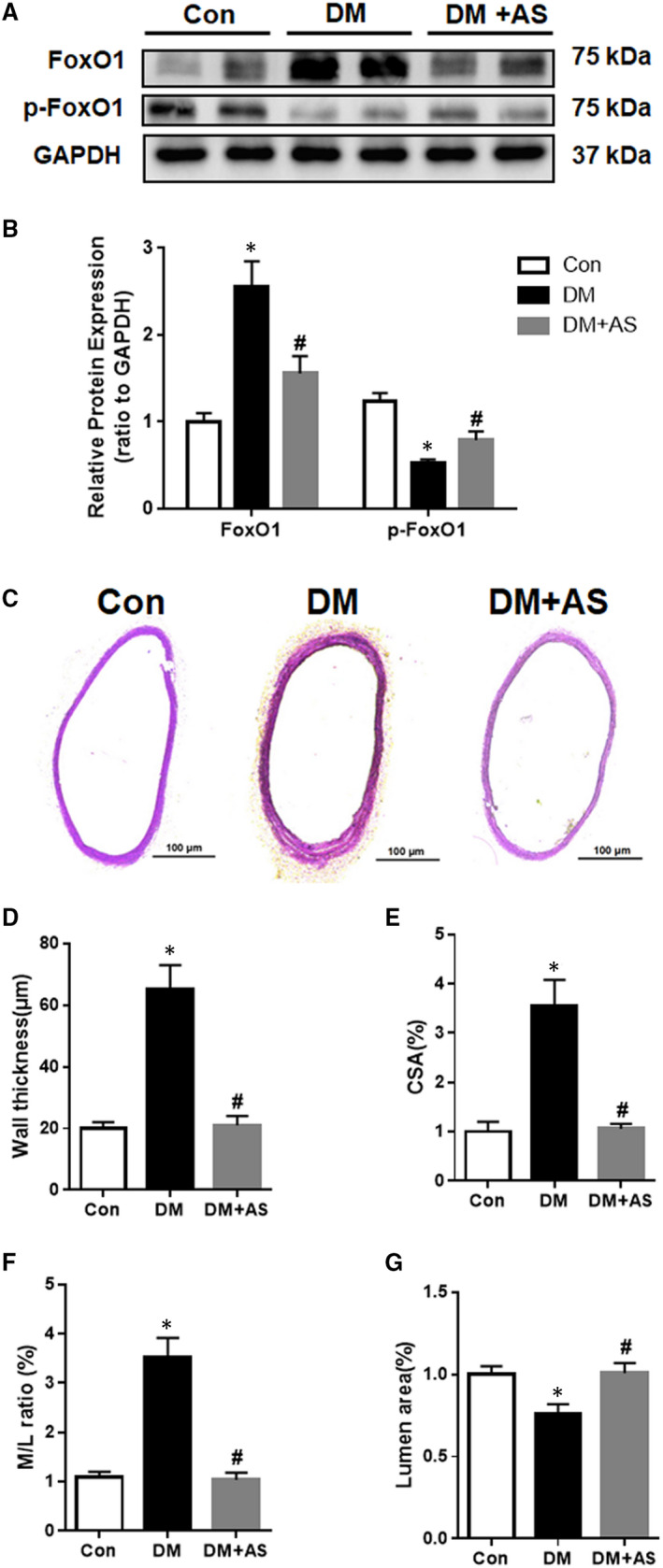
Up‐regulation of FoxO1 is associated with changes in vascular structure in diabetic rat carotid artery. (A) Western blot analysis of FoxO1 and phosphorylated (p)‐FoxO1 protein expression in carotid arteries from STZ (streptozotocin)‐treated rats with/without AS treatment. Protein expression was normalized to housekeeping protein GAPDH. (B) Mean values of normalized protein expression of FoxO1 and phosphorylated (p)‐FoxO1 in carotid arteries from diabetic rats (n = 6) with/without AS treatment. Data are presented as mean ± SD. *P < .05 vs Control; #*P* < .05 vs Diabetes. Con: Control, DM: diabetic rats. DM + AS: diabetic rats with treatment of FoxO1‐selective inhibitor AS (AS1842856). (C) Representative images of haematoxylin and eosin (H&E) staining of carotid artery samples from Sprague‐Dawley rats (100X magnification). (D) Vascular wall thickness. (E) Vascular cross‐sectional area. (F) Media/lumen ratio. (G) Lumen area

We first investigated the expression of FoxO1 in the carotid arteries of Sprague‐Dawley (SD) rats at 8 weeks of the diabetes. Western blot analysis showed that total FoxO1 expression was up‐regulated in the carotid arteries of diabetic rats, which could be significantly attenuated by treatment with AS (a selective FoxO1 inhibitor AS1842856). On the other hand, the level of phosphorylated FoxO1 in the carotid arteries of diabetic rats was significantly reduced as compared to that in the control group (*P* < .05) and AS treatment partially restored the phosphorylated FoxO1 level in diabetic group (*P* < .05, DM + AS vs DM, Figure 1A,B).

Next, the vascular wall structure was assessed by measuring carotid artery wall thickness, carotid medial cross‐sectional area (CSA), media‐to‐lumen ratio (M/L ratio) and lumen areas at 8 weeks after STZ injection (Figure 1C‐G). H&E staining showed that the wall thickness of carotid artery from diabetic rats was significantly greater than that in non‐diabetic control group (Figure 1D, *P* < .05). T1DM was associated with arterial wall hypertrophy, with significant increases in CSA and M/L ratio as compared to those in non‐diabetic control group (Figure 1E‐F, *P* < .05). In addition, the media thickened to encroach on the lumen and resulted in decreased carotid lumen areas in diabetic rats, as compared with non‐diabetic control group (Figure 1G, *P* < .05). AS treatment also prevented carotid wall morphological changes seen in diabetic rats (all *P* < .05 DM + AS vs DM, Figure 1E‐G). These data showed that inward remodelling occurred in the carotid artery in diabetic rats, which was slowed down or prevented by FoxO1 inhibition, suggesting that FoxO1 contributed to the development of adverse vascular remodelling in T1DM.

**FIGURE 2:**
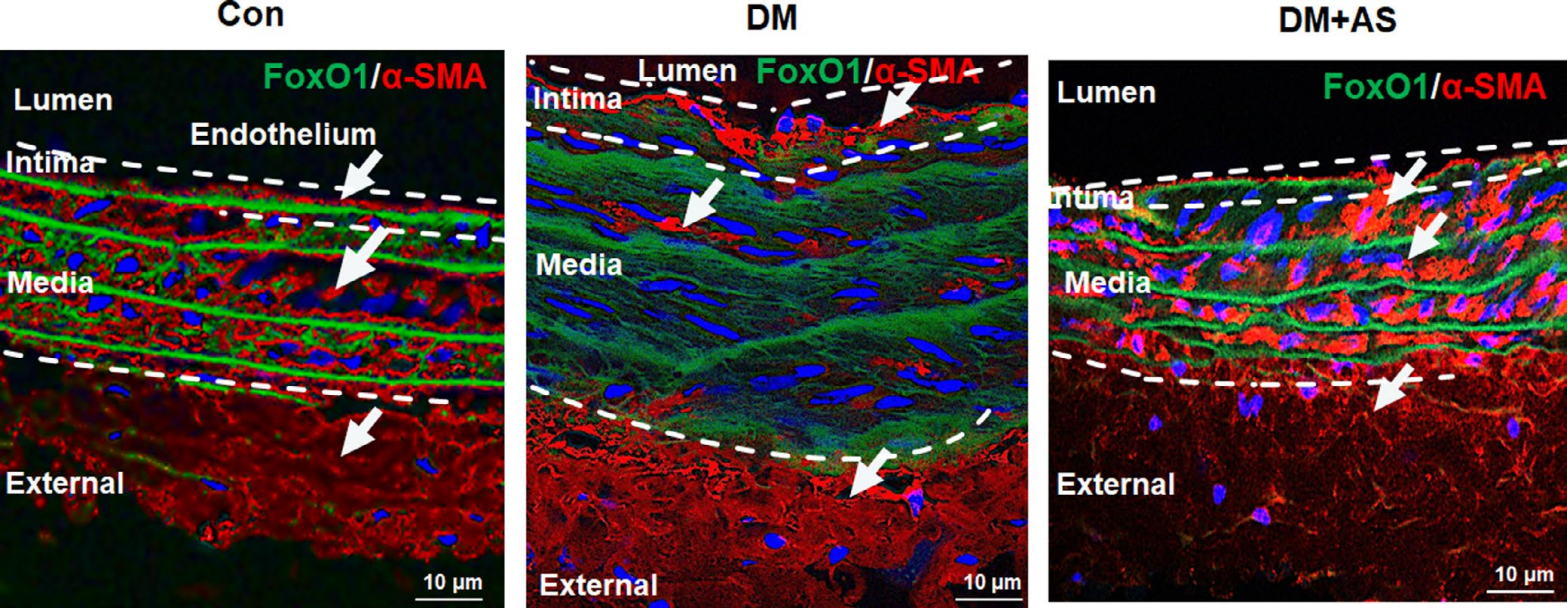
FoxO1 inhibition with AS1842856 restored down‐regulated α‐SMA in the carotid of diabetic rats. Representative immunofluorescence photomicrographs of FoxO1 (green) and α‐smooth muscle actin (α‐SMA, a smooth muscle cell‐specific marker, red) in carotid arteries from STZ‐treated rats with/without AS treatment. Nuclei are counter stained with DAPI (blue). Scale bars: 10 μm. Images are representative of n = 6 carotid arteries per group. Con: Control, DM: diabetic rats. DM + AS: diabetic rats with treatment of FoxO1‐selective inhibitor AS1842856

To further investigate the role of FoxO1 in the pathological process of type 1 diabetes‐induced vascular remodelling, we examined the expression of α‐smooth muscle actin (α‐SMA), a contractile state SMCs‐specific protein marker in carotid arteries. H&E staining showed increased media of carotid occurred in diabetic rats that was consistent with the findings of immunofluorescence staining which showed that α‐SMA expression was down‐regulated in diabetic rat carotid media and was restored by AS treatment (Figure 2).

Thus, the results demonstrated that type 1 diabetes‐induced vascular remodelling was associated with elevated FoxO1 and decreased phosphorylated FoxO1 levels, suggesting that high levels of FoxO1 nuclear translocation stimulated the transcription of certain genes involved in type 1 diabetes‐induced vascular remodelling development.

### 3.2 FoxO1 inhibition ameliorated NLPR3 inflammasome‐dependent inflammation in the carotid artery of type 1 diabetic rats

The mechanism whereby inhibition of FoxO1 attenuated the development of diabetic vascular remoulding also needs to be further investigated. We, thus, observed the blood glucose of rats and the result showed that administration of AS did not affect blood glucose level (Table S2), suggesting that the inhibition of FoxO1 mediated improvement of vascular remodelling in diabetes was independent of blood glucose level.

**FIGURE 3:**
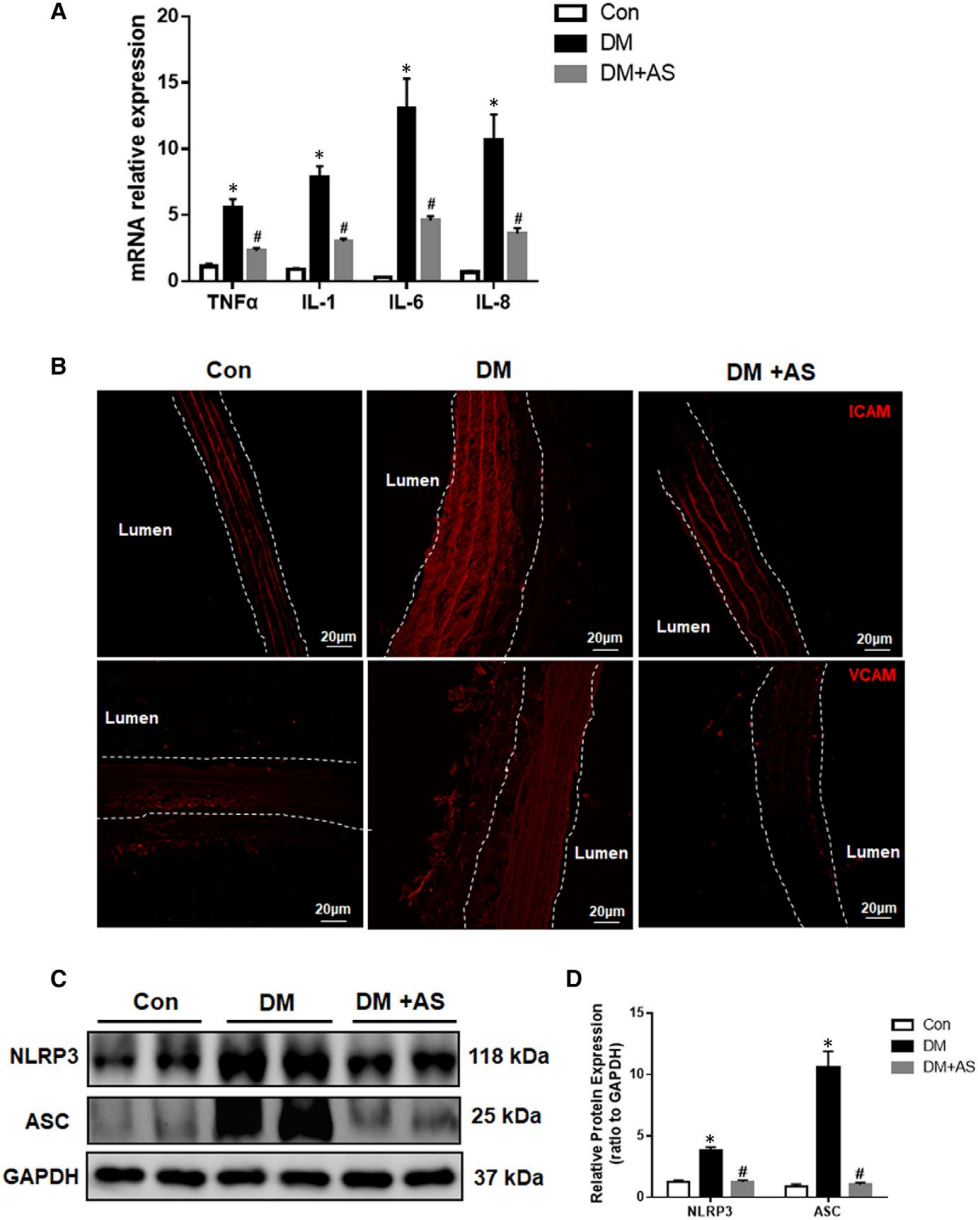
FoxO1 is associated with increased pro‐inflammatory factors in diabetic rat carotid artery. (A) mRNA expression profiles of TNF‐α, IL‐1β, IL‐6 and IL‐8 in carotid arteries derived from real‐time (RT) qPCR from diabetic Sprague‐Dawley rats with/without AS treatment (n = 6). Expression was normalized to housekeeping GAPDH mRNA. (B) Representative immunofluorescence photomicrographs of ICAM‐1 and VCAM‐1 (both in red) in carotid arteries from diabetic rats with/without AS treatment (n = 6, Scale bars: 20 μm). (C) Western blot analysis of NLRP3 (NOD‐like receptor family protein‐3) and ASC (apoptosis‐associated speck‐like protein with a caspase‐recruitment domain) protein expression in carotid arteries from diabetic rats with/without AS treatment. Protein expression was normalized to housekeeping protein GAPDH. (D) Mean values of normalized protein expression of NLRP3 and ASC in carotid arteries from STZ‐treated rats with/without AS treatment (n = 6). Data are presented as mean ± SD. *P < .05 vs Control; #*P* < .05 vs Diabetes. Con: Control, DM: diabetic rats. DM + AS: diabetic rats with treatment of FoxO1‐selective inhibitor AS

Extensive research has identified inflammatory response as a key driver in the initiation and progression of vascular disease within the context of diabetes mellitus, from the early asymptomatic stage of vascular injury to the subsequent clinical manifestation of vascular dysfunction and remodelling in the advanced stage. To investigate whether FoxO1 mediated pro‐inflammatory response in diabetic rats, inflammatory cytokine gene expression was examined by RT‐qPCR. Tumour necrosis factor α (TNF‐α) mRNA expression was up‐regulated in carotid arteries of diabetic rats and was decreased following treatment with AS (Figure 3A). In the meantime, the levels of interleukin (IL)‐1β, IL‐6 and IL‐8 were significantly elevated in carotid arteries in diabetic rats, and such increases were offset by AS (Figure 3A). Given that monocyte attachment is involved in vascular remodelling, Immunofluorescence staining was performed to examine the protein levels of cell adhesion molecules VCAM‐1 and ICAM‐1. The results showed that both VCAM‐1 and ICAM‐1 levels were significantly increased in diabetic rat carotid and were reduced by AS treatment (Figure 3B).

Following the finding that AS reduced diabetes‐induced inflammation, we went on to further investigate the role of NLRP3 (NOD‐like receptor family, pyrin domain containing 3) inflammasome in this pathology, given that NLRP3 has emerged as a central regulator in the inflammatory process that contributes to the aggravation of DM and diabetic complications.^34^ As expected, the expression of key inflammasome‐associated enzymes NLRP3 and ASC (apoptosis‐associated speck‐like protein with a caspase‐recruitment domain) protein levels in diabetic rat carotid artery were significantly increased as compared to those in the control group, and these increases were offset by AS (Figure 3C, D).

### 3.3 Inhibition of FoxO1 prevented cell apoptosis in the carotid artery of diabetic rats

**FIGURE 4:**
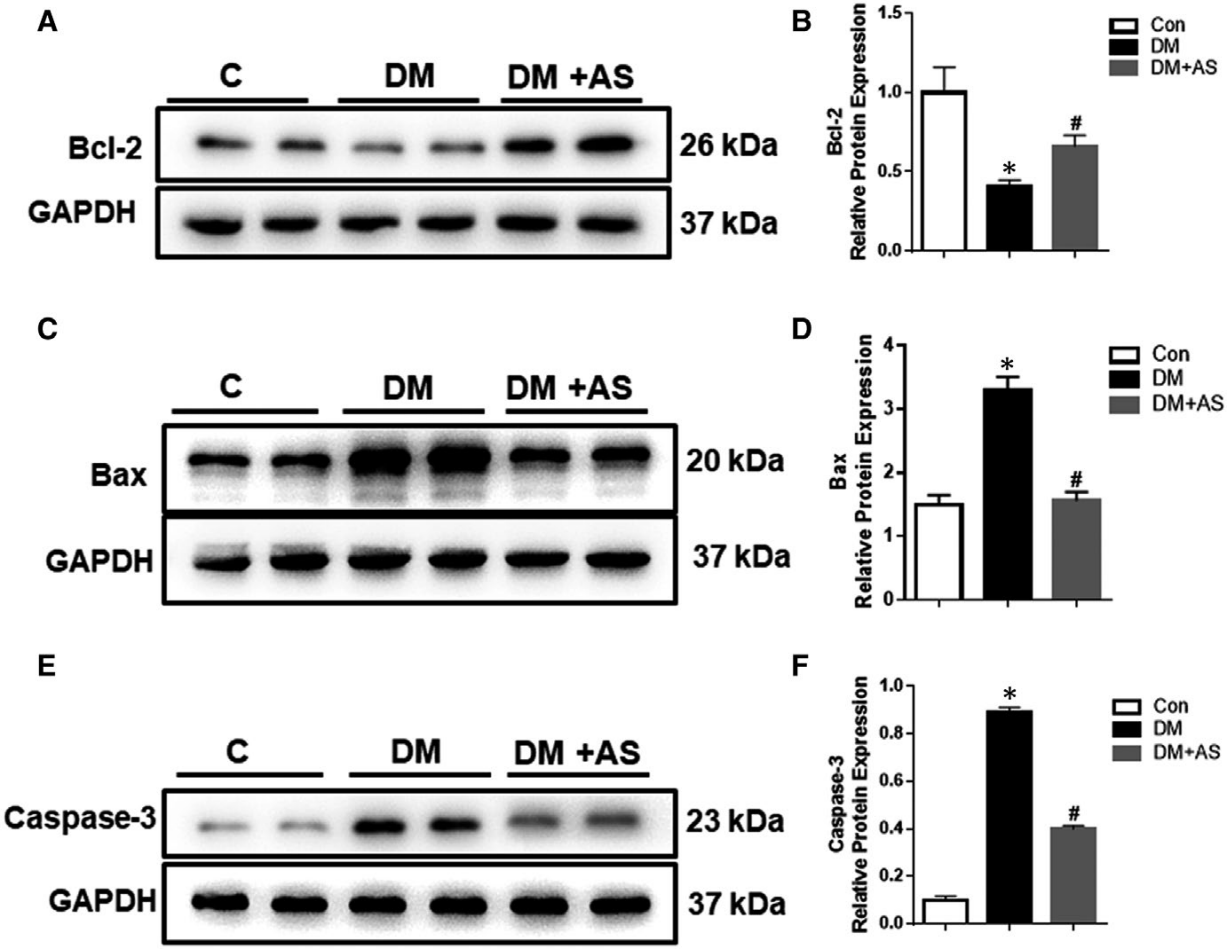
FoxO1 is associated with increased apoptosis in diabetic rat carotid artery. (A‐F) Western blot analysis of Bcl‐2, BaX and caspase‐3 protein expression in carotid arteries from diabetic Sprague‐Dawley rats with/without AS treatment. Protein expression was normalized to housekeeping protein GAPDH. (B, D, F) Mean values of normalized protein expression of Bcl‐2, BaX and caspase‐3 (n = 6). Data are presented as mean ± SD. *P < .05 vs Control; #*P* < .05 vs Diabetes. Con: Control, DM: diabetic rats. DM + AS: diabetic rats with treatment of FoxO1‐selective inhibitor AS

Emerging evidence showed that, in addition to inflammation, vascular proliferation and apoptosis contribute significantly to inward vascular remodelling, with apoptosis localized to the outer periphery which reduces the vessel outer diameter while inward growth decreases lumen diameter despite it contributes to the maintenance of the media volume. Whether apoptosis is a growth‐related compensatory mechanism, or a primary process involved in vessel growth, remains to be clarified. We wondered whether FoxO1 mediated vascular remodelling in diabetes was attributable to the enhancement of apoptosis under hyperglycaemic environment. Our further results showed that the anti‐apoptotic protein Bcl‐2 was significantly down‐regulated (Figure 4A, B, *P* < .05 vs control) whereas the pro‐apoptotic proteins Bax and cleaved Caspase‐3 were significantly up‐regulated in diabetic rats (Figure 4C‐F, *P* < .05 vs control) compared to controls and FoxO1 inhibition reduced those divergences in diabetic rats back towards the control level, along with decreased cleaved Caspase‐3 and Bax and increased Bcl‐2 levels (*P* < .05 vs DM). Thus, our data confirmed the pro‐apoptotic role of FoxO1 in type 1 diabetic rats.

### 3.4 FoxO1 inhibition counteracted the phenotypic switch of SMCs in diabetic rats

**FIGURE 5:**
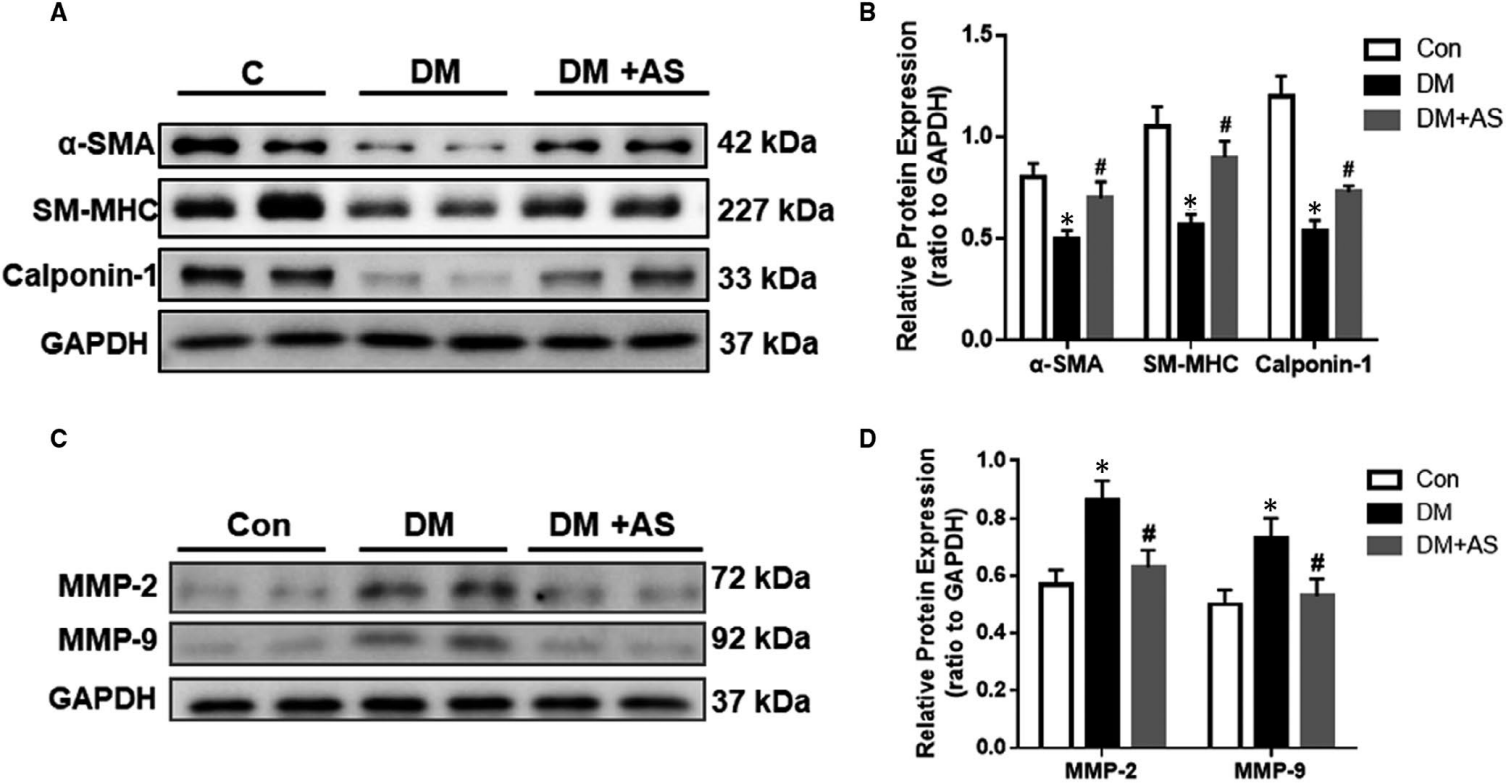
FoxO1 is associated with the synthetic phenotype switch of SMCs and increased expression of MMP‐2 and *MMP‐*9 in diabetic rats. (A) Western blot analysis of α‐SMA, SM‐MHC (smooth muscle myosin heavy chain) and calpoinin‐1 protein expression of carotid arteries from diabetic Sprague‐Dawley rats with/without AS treatment. Protein expression was normalized to housekeeping protein GAPDH. (B) Mean values of normalized protein expression of α‐SMA, SM‐MHC and calpoinin‐1 in carotid arteries from STZ‐treated rats with/without AS treatment. (C) Western blot analysis of MMP (matrix metalloproteinases)‐2 and *MMP‐*9 protein expression in carotid arteries from diabetic Sprague‐Dawley rats with/without AS treatment. Protein expression was normalized to housekeeping protein GAPDH. (D, E) Mean values of normalized protein expression of MMP‐2 and *MMP‐*9 in carotid arteries from diabetic rats with/without AS treatment (n = 6). Con: Control, DM: diabetic rats. DM + AS: diabetic rats with treatment of FoxO1‐selective inhibitor AS1842856

Based on in vivo observation, we postulated that FoxO1 may have contributed to vascular remodelling in diabetes via its impact on cellular phenotypic switching. SMCs exist in a diverse range of phenotypes. In normal mature blood vessels, the predominant phenotype is the quiescent, also known as contractile or differentiated SMCs, whose major function is regulating blood vessel diameter (through vasodilation and vasoconstriction) and blood flow. During pathological vascular remodelling (ex. arteriosclerosis), SMCs with non‐contractile or synthetic phenotype generate intimal vascular lesions. The protein levels of α‐SMA, SM‐MHC and calpoinin‐1, three widely used SMCs differentiation markers, were found to be significantly decreased in diabetic rat carotid arteries, which were all restored towards control levels by AS treatment (Figure 5A, B). Thus, these results indicated that AS treatment reverted the phenotypic switch of SMCs in type 1 diabetic rats and restored it to a contractile state.

### 3.5 Elevation of FoxO1 contributed to the increase in MMP‐2 and MMP‐9 in the carotid arteries of type 1 diabetic rats

As degradation of the extracellular matrix scaffold enables tissue reshaping, participation of specialized enzymes, called matrix metalloproteinases (MMPs), has become the object of interest in relation to vascular remodelling.^20^ Evidence indicates that the major drivers of vascular remodelling, such as haemodynamics, injury, inflammation and oxidative stress, regulate MMP expression and activation. Diffuse wall thickening can be frequently seen in the carotid arteries of type 1 diabetic rats, which is found to be closely associated with MMP‐2 and *MMP‐*9 activation.^35^

In an attempt to further confirm the changes in MMPs expression during inflammation, we measured MMP‐2 and MMP‐9 protein levels. Our results showed that levels of MMP‐2 and MMP‐9 protein expression were both increased in carotid arteries in type 1 diabetic rats, whereas AS treatment decreased those levels back towards control ones (*P* < .05, Figure 5C‐D).

### 3.6 PDK1 regulated FoxO1 nuclear translocation during type 1 diabetes‐induced vascular remodelling

**FIGURE 6:**
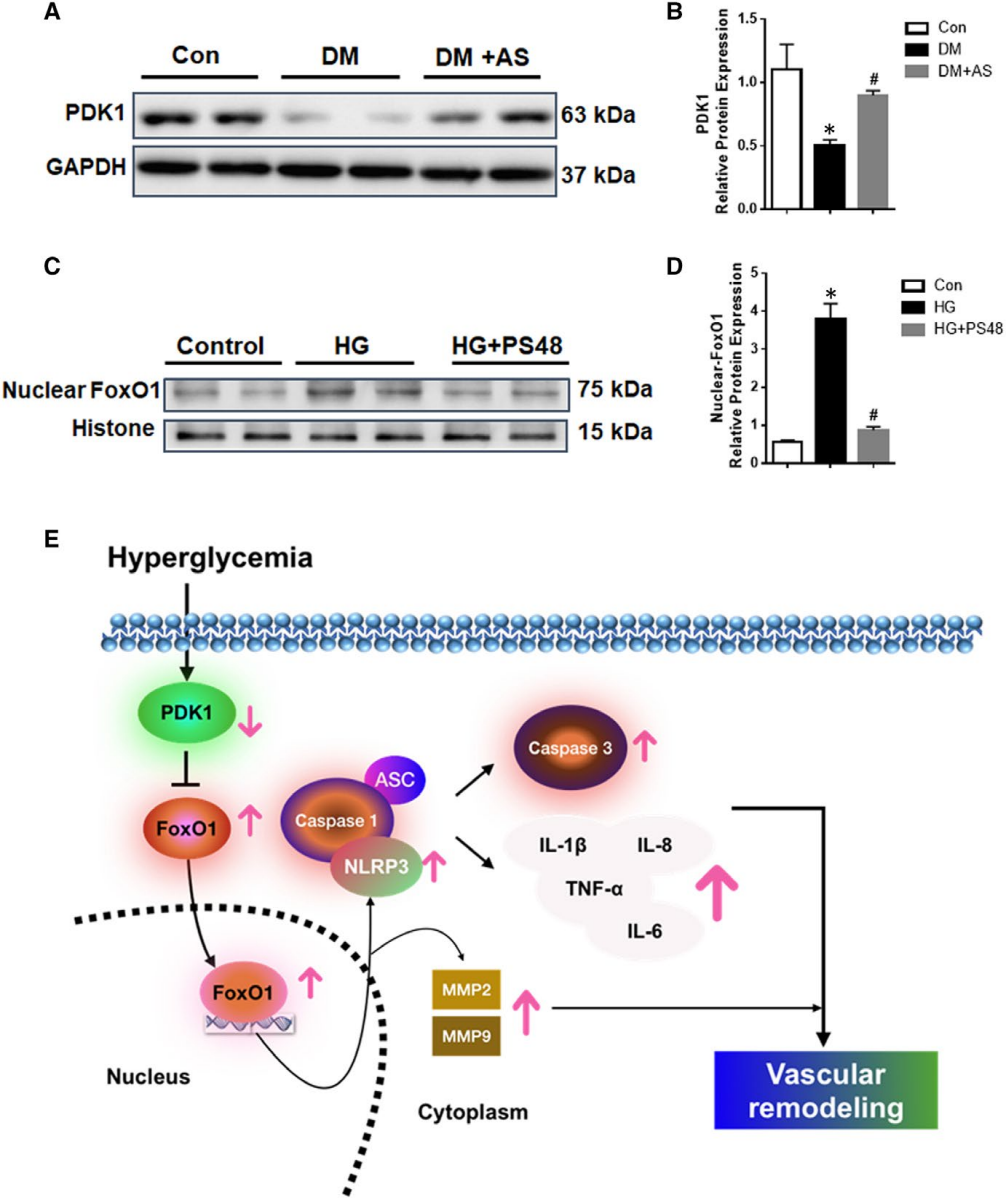
PDK1 is associated with the regulation of FoxO1 translocation and activity in diabetic rat carotid artery and HASMCs. (A) Western blot analysis of PDK1 (phosphoinositide‐dependent kinase) protein expression in carotid arteries from diabetic Sprague‐Dawley rats with/without AS treatment (n = 6). Protein expression was normalized to housekeeping protein GAPDH. (B) Mean values of normalized protein expression for PDK1 *P < .05 vs Control; #*P* < .05 vs Diabetes. (C) Western blot analysis of nuclear FoxO1 expression from HASMCs under high glucose (HG) with/without PS48 (PDK1 activator). Protein expression was normalized to housekeeping protein GAPDH. (D) Mean values of normalized protein expression for nuclear FoxO1. Data are presented as mean ± SD. *P < .05 vs Control; #*P* < .05 vs HG. Control: human aortic smooth muscle cells (HASMCs) in the culture medium. HG: HASMCs in HG medium (30 mM D‐glucose). HG + PS48: HASMCs in HG medium (with treatment of PS48, 5 μM). (E) Graphical abstract. Hyperglycaemia affects the PDK1/FoxO1 pathway which results in FoxO1 activation and the subsequent adverse diabetic vascular remodelling

The 3‐phosphoinositide‐dependent protein kinase 1 (PDK1)/FoxO1 pathway is important in regulating glucose and energy homeostasis,^26^, ^27^ but little is known about its role in type 1 diabetes‐induced vascular remodelling. We, thus, evaluated PDK1 protein level by Western blot in the diabetic rat carotid arteries. The results showed that PDK1 protein expression was markedly decreased in type 1 diabetic rats and restored by AS treatment (Figure 6A, B). To confirm whether PDK 1 was involved in FoxO1 nuclear translocation and the subsequent transcription, nuclear FoxO1 protein expression in cultured HASMCs in the presence of PS48 (PDK1 Activator) and HG were examined by Western blot. The results showed that nuclear protein level of FoxO1 was decreased after application of PS48 (Figure 6C, D), suggesting that PDK1 regulated FoxO1 translocation in HG condition in HASMCs. These findings suggested that high glucose may affect the PDK1‐FoxO1 signalling, resulting in down‐regulation of PDK, along with the subsequent FoxO1 activation and the adverse type 1 diabetes‐induced vascular remodelling (Figure 6E).

## 4 DISCUSSION

The major finding of the current study is that FoxO1 is a novel promoter of vascular remodelling in type1 diabetic rats. We discovered that diabetes increases FoxO1 protein level in rat carotid arteries and the increase in FoxO1 protein is associated with vascular remodelling. Administration of pharmacological FoxO1 inhibitor ameliorated type 1 diabetes‐induced vascular remodelling through decreasing the expression of pro‐inflammatory factors, NLRP3 inflammasome activation, adhesion factors, MMPs and apoptosis, reversing the SMCs phenotypic switching. The underlying mechanism by which FoxO1 pharmacological inhibition reverses the progression of vascular remodelling in T1DM rats includes the activation of PDK1/FoxO1 pathway and the decrease in FoxO1 nuclear translocation. Along with other study,^36^ we found that inhibition of FoxO1 with AS showed a dropping trend in blood glucose level (Table S2), while the differences between groups are not significant (partly because of the limited sample size).

Although great advances in cardiovascular therapy and prevention have reduced diabetes‐related coronary mortality in developed countries, the overall cardiovascular morbidity and mortality are still high in diabetic patients. Moreover, cardiovascular disease (CVD) risk in diabetes remains high even after optimal treatment with statins. Abundant evidence suggests that FoxO1 is an important factor for cardiac metabolic regulation and for maintaining cardiac function. Physiologically, FoxO1 is known to be associated with fasting and cAMP‐induced glycogenolysis and gluconeogenesis,^36^ with respect to the regulation of various metabolic pathways in adapting to fasting and feeding states.^37^ Interestingly, decreased circulating insulin in diabetes is associated with increased FoxO1 activation,^3^ whereas mitochondrial reactive oxygen species (ROS) production was enhanced by altering redox balance and impacting the activity of redox‐sensitive proteins, such as protein kinase C‐ε. Furthermore, ROS reduces insulin signalling and induces subsequent insulin resistance, yielding elevated FoxO1 activation. On the other hand, FoxO1 is involved in angiogenesis through participating in the regulation of endothelial cell metabolism.^9^ Therefore, we investigated the role of FoxO1 in the pathological progress of diabetic vascular complications. In the present study, FoxO1 was consistently found to be highly expressed in carotid arteries, accompanied by hyperglycaemia‐induced vascular remodelling, while FoxO1 inhibitor ameliorated the hyperglycaemia‐induced SMCs decrease in carotid medium of type 1 diabetic rats. This is suggestive of FoxO1 alteration as a major contributing factor to the development of diabetic vascular complications. Of note, diabetic db/db mice with acute administration of AS1842856 drastically decreased fasting plasma glucose.^38^ In our previous study,^16^ AS1842856 treatment reduced mitochondrial ROS content, cardiac apoptosis and improved cardiac performance, but do not have any significant effects on existing hyperglycaemia in STZ‐induced diabetic rats. In this study, the value of random blood glucose rather than fasting plasma glucose was recorded and we found that AS1842856 treatment had no effects on elevated random blood glucose (Table S2) in STZ‐induced diabetic rats model, suggesting the beneficial effects of AS1842856 treatment on diabetic vascular remodelling is not through lowering blood glucose.

Unlike the cardiovascular risk in T1D is mainly driven by hyperglycaemia, the cause of carotid arteries remodelling in T2D is multifactorial, featuring several factors largely absent in T1D such as obesity and hypertension.^39^ Nevertheless, the pathological process involves in carotid arteries remodelling is similar to some extent in both diabetic groups, such as matrix metalloproteinases (MMPs) alteration, inflammatory cell infiltration and smooth muscle cells (SMCs) apoptosis.^19^, ^21^, ^22^ MMPs promote arterial remodelling.^40^ MMP‐associated invasion and de‐differentiation of VSMCs is an essential molecular and cellular event of diffuse IMT (intra medial thickness). Intracellularly activated MMP‐2 cleaves SMCs calponin‐1, a differentiation marker, shifting the cell phenotype to a de‐differentiated state.^41^ Extracellularly activated MMPs cleave collagen and basement membranes, releasing resident VSMCs from a non‐permissive quiescent status to a permissive synthetic state.^42^ Additionally, MMP‐mediated VSMC synthetic phenotypes in vivo also contributes to arterial remodelling.^43^, ^44^ In this study, MMP‐2 and MMP‐9 protein levels were found to be increased in carotid arteries in diabetic rats compared to controls, but decreased in the FoxO1 inhibitor group. Thus, the current study demonstrates the necessity of FoxO1 activation for MMP‐2 and MMP‐9 dependent vascular remodelling. The fact that MMPs are a family of zinc‐dependent endopeptidases highly activated by inflammatory signalling prompted us to investigate the role of inflammation on the vascular remodelling induced by hyperglycaemia.

Inflammation has long been considered as a major risk factor in diabetes, which is associated with development and progression of diabetic complications. Hyperglycaemia‐induced oxidative stress promotes inflammation through increased endothelial cell damage, microvascular permeability and increased release of pro‐inflammatory cytokines, including TNF‐α, interlukin‐1β (IL‐1β) and interlukin‐6 (IL‐6). All these changes ultimately lead to decreased insulin sensitivity and diabetic complications. Hyperglycaemia‐induced FoxO1 plays an important role in inducing pro‐inflammatory cytokines. It is shown that FoxO1 directly binds to the IL‐1β promoter, increasing its expression in macrophages.^45^ Recent studies show that FoxO1 promotes inflammation during diabetes by enhancing TLR4‐mediated signalling.^46^ Our results showed that inflammation was induced in carotid arteries in type 1 diabetic rats, contributing to vascular remodelling that was prevented by FoxO1 inhibitor AS both in vivo and in vitro. These data strengthen the evidence that FoxO1 acts as a key mediator of inflammatory responses during T1DM.

In the context of diabetes, the role of inflammasomes in diabetes‐associated atherosclerosis has attracted only modest attention. A critical role of the NLRP3 inflammasome in the development of diabetic vascular complications has been postulated based on previous studies that reported increases in inflammasome components NLRP3, ASC and IL‐1β both in a diabetic porcine model of atherosclerosis^47^ and STZ‐induced diabetic ApoE KO mice.^48^ Moreover, NLRP3 has been found in macrophage and foam cell cytoplasm, and is associated with cholesterol crystal clefts inside and outside the cells.^49^ Furthermore, NLRP3 gene polymorphisms are strongly correlated with increased risk of macrovascular complications, particularly myocardial infarction, in diabetic patients.^34^ Clinical verification on the role of NLRP3 and NLRP3 activation products in the development of macrovascular complications may serve as a basis to incorporate this inflammasome family as a useful clinical predictor for cardiovascular events in diabetic patients. In addition, previously study found that increased FoxO1 could induced NLRP3 inflammasome expression.^50^ This was confirmed by our data showing increases in NLRP3 inflammasome components, NLRP3 and ASC, in diabetic rat carotid arteries, and these increases were offset by AS treatment. These data strengthen the evidence for FoxO1 as a mediator of inflammasome‐dependent inflammation in diabetes.

The PDK1/FoxO1 pathway is important in regulating glucose and energy homeostasis, but little is known about this pathway in carotid tissue under hyperglycaemic condition. It has reported that PDK1 contribute to increased serotonin‐induced contraction in the carotid arteries of type 2 diabetic rats and the underlying mechanism is still unclear.^51^, ^52^ The insulin signalling has been reported to activate PDK1 in carotid artery.^52^ In this way, plasma insulin level is absolutely deficient in our STZ‐induced type 1 diabetic rats (data not shown), which is consistent with the decreased expression or activity of PDK1 in carotid artery of type 1 diabetic rats in our current study. We further demonstrated that PDK1 was involved in the upstream signalling response to hyperglycaemia. The disrupted PDK1/FoxO1 pathway therefore promoted FoxO1 nuclear translocation for subsequent transcriptional regulation. These data may represent a novel mechanism to explain the role of PDK1 in carotid arteries remodelling of type 1 diabetes.

Collectively, we demonstrated that FoxO1 as a novel trigger for the progression of type 1 diabetes‐induced vascular remodelling via its initiation of NLPR3 inflammasome‐dependent inflammation. The interaction between PDK1/FoxO1/NLPR3 inflammasome may have important therapeutic implication for diabetic cardiovascular diseases.

## CONFLICT OF INTEREST

The authors declared that they have no conflicts of interest to this work.

## AUTHOR CONTRIBUTIONS

**Jingjin Liu:** Conceptualization (equal); Data curation (lead); Investigation (lead); Methodology (equal); Validation (equal); Writing‐original draft (lead); Writing‐review & editing (equal). **Xiang Xie:** Conceptualization (equal); Data curation (equal); Formal analysis (equal); Investigation (equal); Methodology (equal); Writing‐original draft (equal); Writing‐review & editing (supporting). **Dan Yan:** Formal analysis (equal); Investigation (equal); Methodology (equal). **Yongshun Wang:** Formal analysis (equal). **Hongbin Yuan:** Formal analysis (equal). **Yin Cai:** Data curation (supporting); Formal analysis (supporting); Investigation (supporting); Methodology (supporting); Writing‐original draft (supporting); Writing‐review & editing (supporting). **Jierong Luo:** Investigation (supporting); Methodology (supporting). **Aimin Xu:** Software (supporting); Writing‐review & editing (supporting). **Yu Huang:** Writing‐review & editing (supporting). **Chi‐Wai Cheung:** Methodology (supporting); Project administration (supporting); Writing‐original draft (supporting). **Michael G. Irwin:** Writing‐review & editing (equal). **zhengyuan xia:** Conceptualization (lead); Funding acquisition (lead); Writing‐original draft (supporting); Writing‐review & editing (lead).

## Supporting information

Table S1‐S2

## References

[1] Nakae J , Biggs WH , Kitamura T , et al. Regulation of insulin action and pancreatic β‐cell function by mutated alleles of the gene encoding forkhead transcription factor Foxo1. Nat Genet. 2002;32(2):245. 12219087 10.1038/ng890

[2] Altomonte J , Cong L , Harbaran S , et al. Foxo1 mediates insulin action on apoC‐III and triglyceride metabolism. J Clin Investig. 2004;114(10):1493‐1503. 15546000 10.1172/JCI19992 PMC525736

[3] Gross D , Van den Heuvel A , Birnbaum M . The role of FoxO in the regulation of metabolism. Oncogene. 2008;27(16):2320. 18391974 10.1038/onc.2008.25

[4] Wang Y , Zhou Y , Graves DT . FOXO transcription factors: their clinical significance and regulation. Biomed Res Int. 2014;2014. 10.1155/2014/925350 PMC4016844 24864265

[5] Karbasforooshan H , Karimi G . The role of SIRT1 in diabetic retinopathy. Biomed Pharmacother. 2018;97:190‐194. 29091865 10.1016/j.biopha.2017.10.075

[6] Li W , Du M , Wang Q , et al. FoxO1 promotes mitophagy in the podocytes of diabetic male mice via the PINK1/Parkin pathway. Endocrinology. 2017;158(7):2155‐2167. 28505239 10.1210/en.2016-1970

[7] Matsumoto M , Han S , Kitamura T , Accili D . Dual role of transcription factor FoxO1 in controlling hepatic insulin sensitivity and lipid metabolism. J Clin Investig. 2006;116(9):2464‐2472. 16906224 10.1172/JCI27047 PMC1533874

[8] Tsuzuki K , Itoh Y , Inoue Y , Hayashi H . TRB1 negatively regulates gluconeogenesis by suppressing the transcriptional activity of FOXO1. FEBS Lett. 2018;593:369‐380. 10.1002/1873-3468.13314 30556236

[9] Potente M , Urbich C , Sasaki KI , et al. Involvement of Foxo transcription factors in angiogenesis and postnatal neovascularization. J Clin Investig. 2005;115(9):2382‐2392. 16100571 10.1172/JCI23126 PMC1184037

[10] Stitt TN , Drujan D , Clarke BA , et al. The IGF‐1/PI3K/Akt pathway prevents expression of muscle atrophy‐induced ubiquitin ligases by inhibiting FOXO transcription factors. Mol Cell. 2004;14(3):395‐403. 15125842 10.1016/s1097-2765(04)00211-4

[11] Nakae J , Kitamura T , Kitamura Y , et al. The forkhead transcription factor Foxo1 regulates adipocyte differentiation. Dev Cell. 2003;4(1):119‐129. 12530968 10.1016/s1534-5807(02)00401-x

[12] Zhang H , Ge S , He K , et al. FoxO1 inhibits autophagosome‐lysosome fusion leading to endothelial autophagic‐apoptosis in diabetes. Cardiovasc Res. 2019;115(14):2008‐2020. 10.1093/cvr/cvz014 30689742

[13] Behl Y , Krothapalli P , Desta T , et al. FOXO1 plays an important role in enhanced microvascular cell apoptosis and microvascular cell loss in type 1 and type 2 diabetic rats. Diabetes. 2009;58(4):917‐925. 19168598 10.2337/db08-0537 PMC2661587

[14] Alblowi J , Kayal RA , Siqueria M , et al. High levels of tumor necrosis factor‐α contribute to accelerated loss of cartilage in diabetic fracture healing. Am J Pathol. 2009;175(4):1574‐1585. 19745063 10.2353/ajpath.2009.090148 PMC2751554

[15] Kandula V , Kosuru R , Li H , et al. Forkhead box transcription factor 1: role in the pathogenesis of diabetic cardiomyopathy. Cardiovasc Diabetol. 2016;15:44. 26956801 10.1186/s12933-016-0361-1 PMC4784400

[16] Yan D , Cai Y , Luo J , et al. FOXO1 contributes to diabetic cardiomyopathy via inducing imbalanced oxidative metabolism in type 1 diabetes. J Cell Mol Med. 2020;24(14):7850‐7861. 32450616 10.1111/jcmm.15418 PMC7348139

[17] Epstein FH , Gibbons GH , Dzau VJ . The emerging concept of vascular remodeling. N Engl J Med. 1994;330(20):1431‐1438. 8159199 10.1056/NEJM199405193302008

[18] Westerhof N , et al. Mechanotransduction and vascular remodeling Snapshots of Hemodynamics. Springer; 2019:245‐255.

[19] Jansen F , Zietzer A , Stumpf T , et al. Endothelial microparticle‐promoted inhibition of vascular remodeling is abrogated under hyperglycaemic conditions. J Mol Cell Cardiol. 2017;112:91‐94. 28919327 10.1016/j.yjmcc.2017.09.004

[20] Creemers EE , Cleutjens JP , Smits JF , Daemen MJ . Matrix metalloproteinase inhibition after myocardial infarction: a new approach to prevent heart failure? Circ Res. 2001;89(3):201‐210. 11485970 10.1161/hh1501.094396

[21] Burke AP , Kolodgie FD , Zieske A , et al. Morphologic findings of coronary atherosclerotic plaques in diabetics: a postmortem study. Arterioscler Thromb Vasc Biol. 2004;24(7):1266‐1271. 15142859 10.1161/01.ATV.0000131783.74034.97

[22] Fukumoto H , Naito Z , Asano G , et al. Immunohistochemical and morphometric evaluations of coronary atherosclerotic plaques associated with myocardial infarction and diabetes mellitus. J Atheroscler Thromb. 1998;5(1):29‐35. 10077455 10.5551/jat1994.5.29

[23] Arencibia JM , Pastor‐Flores D , Bauer AF , et al. AGC protein kinases: from structural mechanism of regulation to allosteric drug development for the treatment of human diseases. Biochim Biophys Acta. 2013;1834(7):1302‐1321. 23524293 10.1016/j.bbapap.2013.03.010

[24] Calleja V , Laguerre M , de las Heras‐Martinez G , et al. Acute regulation of PDK1 by a complex interplay of molecular switches. Biochem Soc Trans. 2014;42(5):1435‐1440. 25233428 10.1042/BST20140222

[25] Najafov A , Sommer E , Axten J , et al. Characterization of GSK2334470, a novel and highly specific inhibitor of PDK1. Biochem J. 2011;433(2):357‐369. 21087210 10.1042/BJ20101732

[26] Wick KL , Liu F . A new molecular target of insulin action: regulating the pivotal PDK1. Curr Drug Targets Immune Endocr Metabol Disord. 2001;1(3):209‐221. 12477287 10.2174/1568008013341082

[27] Tawaramoto K , Kotani KO , Hashiramoto M , et al. Ablation of 3‐phosphoinositide‐dependent protein kinase 1 (PDK1) in vascular endothelial cells enhances insulin sensitivity by reducing visceral fat and suppressing angiogenesis. Mol Endocrinol. 2012;26(1):95‐109. 22108800 10.1210/me.2010-0412 PMC5417157

[28] Nagashima T , Shigematsu N , Maruki R , et al. Discovery of novel Foxo1 inhibitors for treating type 2 diabetes: improvement of fasting glycemia in diabetic db/db mice. Mol Pharmacol. 2010;78(5):961‐970. 20736318 10.1124/mol.110.065714

[29] Diep C , Charles N , Blake Gilks C , et al. Progesterone receptors induce FOXO1‐dependent senescence in ovarian cancer cells. Cell Cycle. 2013;12(9):1433‐1449. 23574718 10.4161/cc.24550 PMC3674071

[30] Li H , Yao W , Liu Z , et al. Hyperglycemia abrogates ischemic postconditioning cardioprotection by impairing AdipoR1/Caveolin‐3/STAT3 signaling in diabetic rats. Diabetes. 2015;65(4):942‐955. 26718505 10.2337/db15-0782

[31] Xu W‐N , Zheng HL , Yang RZ , Jiang LS , Jiang SD . HIF‐1α regulates glucocorticoid‐induced osteoporosis through PDK1/AKT/mTOR signaling pathway. Front Endocrinol. 2020;10:922. 10.3389/fendo.2019.00922 PMC6997475 32047474

[32] Cai Y , Sukhova GK , Wong HK , et al. Rap1 induces cytokine production in pro‐inflammatory macrophages through NFκB signaling and is highly expressed in human atherosclerotic lesions. Cell Cycle. 2015;14(22):3580‐3592. 26505215 10.1080/15384101.2015.1100771 PMC4825742

[33] Wu Q , Wang T , Chen S , et al. Cardiac protective effects of remote ischaemic preconditioning in children undergoing tetralogy of fallot repair surgery: a randomized controlled trial. Eur Heart J. 2017;39(12):1028‐1037. 10.1093/eurheartj/ehx030 PMC6018784 28329231

[34] Klen J , Goričar K , Janež A , et al. NLRP3 inflammasome polymorphism and macrovascular complications in type 2 diabetes patients. J Diabetes Res. 2015. 10.1155/2015/616747 PMC4530261 26273672

[35] Galis ZS , Khatri JJ . Matrix metalloproteinases in vascular remodeling and atherogenesis: the good, the bad, and the ugly. Circ Res. 2002;90(3):251‐262. 11861412

[36] Matsumoto M , Pocai A , Rossetti L , DePinho RA , Accili D . Impaired regulation of hepatic glucose production in mice lacking the forkhead transcription factor Foxo1 in liver. Cell Metab. 2007;6(3):208‐216. 17767907 10.1016/j.cmet.2007.08.006

[37] Zhang W , Patil S , Chauhan B ,, et al. FoxO1 regulates multiple metabolic pathways in the liver effects on gluconeogenic, glycolytic, and lipogenic gene expression. J Biol Chem. 2006;281(15):10105‐10117. 16492665 10.1074/jbc.M600272200

[38] Nagashima T , Shigematsu N , Maruki R , et al. Discovery of novel forkhead box O1 inhibitors for treating type 2 diabetes: improvement of fasting glycemia in diabetic db/db. Mice. 2010;78(5):961‐970. 10.1124/mol.110.065714 20736318

[39] Yahagi K , Kolodgie FD , Lutter C , et al. Pathology of human coronary and carotid artery atherosclerosis and vascular calcification in diabetes mellitus. Arterioscler Thromb Vasc Biol. 2017;37(2):191‐204. 27908890 10.1161/ATVBAHA.116.306256 PMC5269516

[40] Wang M , Kim SH , Monticone RE , et al. Matrix metalloproteinases promote arterial remodeling in aging, hypertension, and atherosclerosis. Hypertension. 2015;65(4):698‐703. 25667214 10.1161/HYPERTENSIONAHA.114.03618 PMC4359070

[41] Castro MM , Cena J , Cho WJ , et al. Matrix metalloproteinase‐2 proteolysis of calponin‐1 contributes to vascular hypocontractility in endotoxemic rats. Arterioscler Thromb Vasc Biol. 2012;32(3):662‐668. 22199370 10.1161/ATVBAHA.111.242685

[42] Johnson JL . Matrix metalloproteinases: influence on smooth muscle cells and atherosclerotic plaque stability. Expert Rev Cardiovasc Ther. 2007;5(2):265‐282. 17338671 10.1586/14779072.5.2.265

[43] Austin KM , Nguyen N , Javid G , et al. Noncanonical matrix metalloprotease‐1‐protease‐activated receptor‐1 signaling triggers vascular smooth muscle cell dedifferentiation and arterial stenosis. J Biol Chem. 2013;288(32):23105‐23115. 23814055 10.1074/jbc.M113.467019 PMC3743483

[44] Moe KT , Naylynn TM , Yin NO , et al. Tumor necrosis factor‐α induces aortic intima‐media thickening via perivascular adipose tissue inflammation. J Vasc Res. 2013;50(3):228‐237. 23711955 10.1159/000350542

[45] Su D , Coudriet GM , Kim DH , et al. FoxO1 links insulin resistance to proinflammatory cytokine IL‐1β production in macrophages. Diabetes. 2009;58(11):2624‐2633. 19651810 10.2337/db09-0232 PMC2768186

[46] Fan WuQiang , Morinaga H , Kim JJ , et al. FoxO1 regulates Tlr4 inflammatory pathway signalling in macrophages. EMBO J. 2010;29(24):4223‐4236. 21045807 10.1038/emboj.2010.268 PMC3018786

[47] Li YU , Xu S , Jiang B , et al. Activation of sterol regulatory element binding protein and NLRP3 inflammasome in atherosclerotic lesion development in diabetic pigs. PLoS One. 2013;8(6):e67532. 23825667 10.1371/journal.pone.0067532 PMC3692453

[48] Leng W , Ouyang X , Lei X , et al. The SGLT‐2 inhibitor dapagliflozin has a therapeutic effect on atherosclerosis in diabetic ApoE−/− mice. Mediators Inflamm. 2016;2016. 10.1155/2016/6305735 PMC5220517 28104929

[49] Shi X , Xie W‐L , Kong W‐W , et al. Expression of the NLRP3 inflammasome in carotid atherosclerosis. J Stroke Cerebrovasc Dis. 2015;24(11):2455‐2466. 26381780 10.1016/j.jstrokecerebrovasdis.2015.03.024

[50] Kim DH , Kim SM , Lee B , et al. Effect of betaine on hepatic insulin resistance through FOXO1‐induced NLRP3 inflammasome. J Nutr Biochem. 2017;45:104‐114. 28499186 10.1016/j.jnutbio.2017.04.014

[51] Watanabe S , Matsumoto T , Taguchi K , et al. Relationship between PDK1 and contraction in carotid arteries in Goto‐Kakizaki rat, a spontaneous type 2 diabetic animal model. Can J Physiol Pharmacol. 2017;95(4):459‐462. 28118732 10.1139/cjpp-2016-0372

[52] Watanabe S , Matsumoto T , Oda M , et al. Insulin augments serotonin‐induced contraction via activation of the IR/PI3K/PDK1 pathway in the rat carotid artery. Pflugers Arch. 2016;468(4):667‐677. 26577585 10.1007/s00424-015-1759-4

