# Supplementary material for: Up‐regulation of FoxO1 contributes to adverse vascular remodelling in type 1 diabetic rats
Source: J Cell Mol Med. 2020 Oct 27;24(23):13727–38. doi: 10.1111/jcmm.15935 (PMC7754018; doi:10.1111/jcmm.15935)
Supplement: Supplementary file 1 — Table S1‐S2 [file JCMM-24-13727-s001.docx]

Table S1: List of oligonucleotides used for RT-qPCR

| Primer | Sequence (5′-3′) |
| --- | --- |
| TNF-α  IL-1β | Forward: TCCCAACAAGGAGGAGAAGTTCC  Reverse: GGCAGCCTTGTCCCTTGAAGAGA  Forward: CTTCATGGTCCGTGGTACCGCCCTGGAGTCT  Reverse: TGCCCCAGGGCATGGCACCAATCACCCTCTGAC |
| IL-6 | Forward: CTGATTGGAAACCTTATTAAG  Reverse: CTGGTAGTATTACCTTCTTCA |
| IL-8 | Forward: GTAGCGAAGGACCTGTCAAACATTGGAAGATCA  Reverse: CAACTCAGTGACCTTGCCTCCTCATGGTAATAC |
| GAPDH | Forward: AGGTCGGTGTGAACGGATTTG Reverse: TGTAGACCATGTAGTTGAGGTC |

Table S2: Effects of AS treatment on metabolic parameters

| Parameters | CON | DM | DM + AS |
| --- | --- | --- | --- |
| Food intake (g/kg/day)  Water consumption (ml/kg/day)  Body weight (g) | 73.5 ±1.6  115.3 ± 8.9  457.1±45.8 | 187.7 ± 21.2**  896.9 ± 108.2**  298.2±24.1 | 168.3 ± 22.6**  940.1 ± 162.2**  309.3±25.8 |
| Plasma glucose (mM) | 7.9 ± 0.7 | 32.1 ± 2.4** | 31.8 ± 2.9** |
| Heart/Body ratio (g/kg) | 2.45 ± 0.14 | 3.17 ± 0.34** | 1.55 ± 0.05^##^ |
|  | | | |

**Table**. Food intake and water consumption were the average value of the last week of the study when 50mg/kg AS1842856 was administered to the subgroup of diabetic rats. Body weight, plasma glucose and triglyceride were determined at the end of the experiment. CON indicates control group. DM indicates diabetic group and DM+50mg/kg AS indicates a subgroup of diabetes treated with 50mg/kg AS1842856. All values are expressed as mean ± S.E.M. n=7 per group. **, p<0.01 vs. CON, ##, p<0.01 vs. DM.
